# Associations Between Oncology Outreach and Patient‐Sharing Measures of Care Coordination

**DOI:** 10.1002/cam4.70489

**Published:** 2024-12-10

**Authors:** Bruno T. Scodari, Andrew P. Schaefer, Nirav S. Kapadia, A. James O'Malley, Erika L. Moen

**Affiliations:** ^1^ Department of Biomedical Data Science Geisel School of Medicine at Dartmouth Lebanon New Hampshire USA; ^2^ The Dartmouth Institute for Health Policy and Clinical Practice Geisel School of Medicine at Dartmouth Lebanon New Hampshire USA; ^3^ Dartmouth Cancer Center Geisel School of Medicine at Dartmouth Lebanon New Hampshire USA; ^4^ Department of Radiation Oncology and Applied Sciences Geisel School of Medicine at Dartmouth Lebanon New Hampshire USA

**Keywords:** care coordination, oncology outreach, patient‐sharing network

## Abstract

**Background:**

Oncology outreach is a common strategy for addressing cancer workforce shortages, where traveling oncologists commute across clinical settings to extend their services. Despite its known benefits specifically for rural patients, oncology outreach reallocates physician resources to satellite clinics and may negatively impact the coordination of cancer care.

**Methods:**

In this retrospective study, we identified patients with incident breast, colorectal, and lung cancers from 2016–2019 nationwide Medicare claims and linked them to oncologists using Part B. We considered encounters occurring outside the physician's primary hospital service area as “outreach visits” and calculated the proportion of outreach visits by oncology specialty for contiguous US hospital referral regions (HRRs) using 2016–2017 claims. We constructed a nationwide physician patient‐sharing network from 2018–2019 claims and computed median *care density—*a measure of physician team familiarity—and *local transitivity—*a measure of physician cohesion/clustering—for each HRR as proxies for care coordination. Generalized linear models were used to explore the associations between oncology outreach and care coordination measures at the HRR level.

**Results:**

We found that HRRs with high medical oncology outreach were associated with 16% decreases in care density (95% CI: 5–25) and 4% decreases in local transitivity (95% CI: 1–8) compared to HRRs with low medical oncology outreach. HRRs with high radiation and surgical oncology outreach were not associated with network‐based measures of care coordination.

**Conclusions:**

While medical oncology outreach increases access for underserved patient populations, it potentially fragments care delivery across clinical settings. Health systems may consider this trade‐off to inform decisions concerning the implementation of outreach programs or policies aimed at hedging against fragmentation in markets with active outreach arrangements.

## 1 Introduction

Oncology outreach is a commonly used strategy for addressing cancer workforce shortages [1]. Under the outreach model, traveling medical, radiation, and surgical oncologists (herein referred to as “oncologists”) commute to secondary clinics to extend specialized care on a recurring basis [2, 3, 4]. Mounting evidence suggests that oncology outreach is particularly useful for improving the accessibility [4, 5, 6], timeliness [7], and utilization [8] of cancer care for patients living in rural communities. As a result, several rural health systems have adopted oncology outreach arrangements [5, 9, 10, 11]. While the implementation of oncology outreach may alleviate some of the barriers to specialized care often experienced by patients who reside in underserved communities, it comes at the cost of modifying existing physician relationships by requiring oncologists to apportion their time between primary and satellite clinics. Examining how oncology outreach impacts care coordination at the market level is critical given that these oncologists bridge geographically dispersed teams [12].

Care coordination has been previously defined as “the deliberate organization of patient care activities between two or more participants (including the patient) involved in a patient's care to facilitate the appropriate delivery of healthcare services.” [13] Despite difficulty in capturing this definition, the structure of physician relationships can provide insights into the coordination of cancer care. An effective way of inferring physician relationships is to connect physicians who share patients using administrative claims, resulting in a “patient‐sharing network.” [14, 15] Recent advancements in network analysis have leveraged these networks to develop various measures that quantify structural elements of care coordination, which have been previously associated with patient access, quality, and cost‐related outcomes [16, 17, 18, 19, 20, 21]. For instance, *care density* reflects the extent of patient‐sharing among a patient's team of treating physicians. Patients with high care density are treated by physicians who share many patients among each other, indicating a high level of team familiarity, and are thought to be better positioned to receive coordinated care. Another useful network measure is *local transitivity*, which represents a physician's propensity to share patients with physicians who are also connected with each other (i.e., a friend of a friend is a friend of mine). Physicians with high local transitivity often form cohesive clusters/cliques and are thought to be better positioned to provide coordinated care. As these network‐based measures capture distinct patient‐ and physician‐level elements of care coordination, they provide a novel opportunity to (1) analyze different elements of care coordination across stakeholders and (2) study the general implications of oncology outreach on care coordination. Such research may help guide future oncology outreach policy solutions that seek to jointly optimize both rural access to cancer care and the coordination of cancer care across the system.

In this retrospective study, we aimed to quantify associations between oncology outreach levels and these network‐based care coordination measures among the 304 contiguous US hospital referral regions (HRRs). We aggregated network measures to the HRR level by assigning patients and physicians to a single HRR (using the plurality of their claims) and calculating the medians of the resulting distributions. We selected HRRs as ecological units of observation, as they group together geographic regions that span common referral networks and are thought to approximate large health systems [22]. We hypothesized that HRRs with higher oncology outreach would be associated with decreased care coordination measures, given their greater rates of physician reallocation.

## 2 Methods

### 2.1 Data and Approvals

The data used in this retrospective study are not publicly available due to a data use agreement with the Centers for Medicare and Medicaid Services (CMS). Dartmouth College's institutional review board approved all study protocols and issued a waiver for obtaining informed consent from human subjects prior to commencement of the study.

### 2.2 Study Cohort

We identified incident patients with a biopsy for breast, colorectal, and lung cancers in 2016–2019 followed by two cancer diagnosis codes in the following 12 months using 100% fee‐for‐service Medicare claims (Table S1) [23]. We excluded patients who had a cancer diagnosis code in the 12 months prior to biopsy, were younger than 66 years or older than 99 years of age at biopsy, were not continuously enrolled in Medicare Parts A and B in the 12 months prior and following biopsy, received multiple cancer diagnoses, or had a missing or non‐US ZIP code. Breast, colorectal, and lung cancers were selected due to their prevalence in the population and dependence on multidisciplinary cancer teams [24].

### 2.3 HRR Demographic Variables

We calculated the proportion of Medicare beneficiaries who were Black, Hispanic, male, Medicaid eligible, living in a rural (non‐urban) area, and living in a low‐income area by HRR. To do so, beneficiary race, sex, and Medicaid eligibility were identified in the CMS Master Beneficiary Summary file. Annual residential ZIP code was used to assign the four‐tiered rural categorization (according to secondary rural–urban commuting codes) and determine whether the beneficiary lived in a low‐income area, defined as a ZIP code with > 20% of individuals below the poverty line per the American Community Survey [25, 26]. Given each beneficiary's residential ZIP code and using the Dartmouth Atlas' ZIP code to HRR crosswalk, we aggregated these measures to the HRR level and computed the proportions of interest [22].

### 2.4 Oncologist Per Capita Measures

In addition to these HRR‐level attributes, we calculated oncologist per capita measures using 2016–2019 claims. To do so, we linked the patients in the study cohort to physicians who provided care to them in the 3 months prior to and 12 months following biopsy, and identified their specialty (i.e., medical, radiation, or surgical oncology) using information from the National Plan and Provider Enumeration System (Table S2) [27]. We calculated the number of oncologists for every 100 study cohort patients within each HRR, where physicians and patients were attributed to HRRs based on the plurality of their claims (i.e., the HRR where they had the greatest number of claims). We calculated these oncologist per capita measures separately for each oncology specialty and adjusted for all three variables during statistical modeling, as an HRR's ability to provide oncology outreach and coordinate cancer care was hypothesized to be dependent on the availability of all specialists.

### 2.5 Oncology Outreach

As in prior studies, we defined an oncology outreach visit as the provision of cancer care by an oncologist operating outside of their primary hospital service area (HSA) [4, 7]. Each outreach visit was determined at the encounter level and attributed to the HSA/HRR corresponding to the claim's billing ZIP code. The exposure of interest was the proportion of oncology outreach visits occurring in an HRR in years 2016–2017. We calculated the proportion of outreach visits separately for each oncology specialty, resulting in three exposure variables. We binned each exposure variable into tertiles given their right‐skewed distributions and to align with methodology from prior research (Figure S1A) [17, 18, 19, 21].

### 2.6 Network Assembly

We constructed a nationwide physician patient‐sharing network by connecting oncologists (of any specialty) who shared patients in years 2018–2019. In this network, the nodes represented individual oncologists, lines connecting oncologists indicated existing professional relationship (“ties”), and the “edge weights” of the ties represented the number of shared patients between oncologists.

### 2.7 Outcomes

**FIGURE 1:**
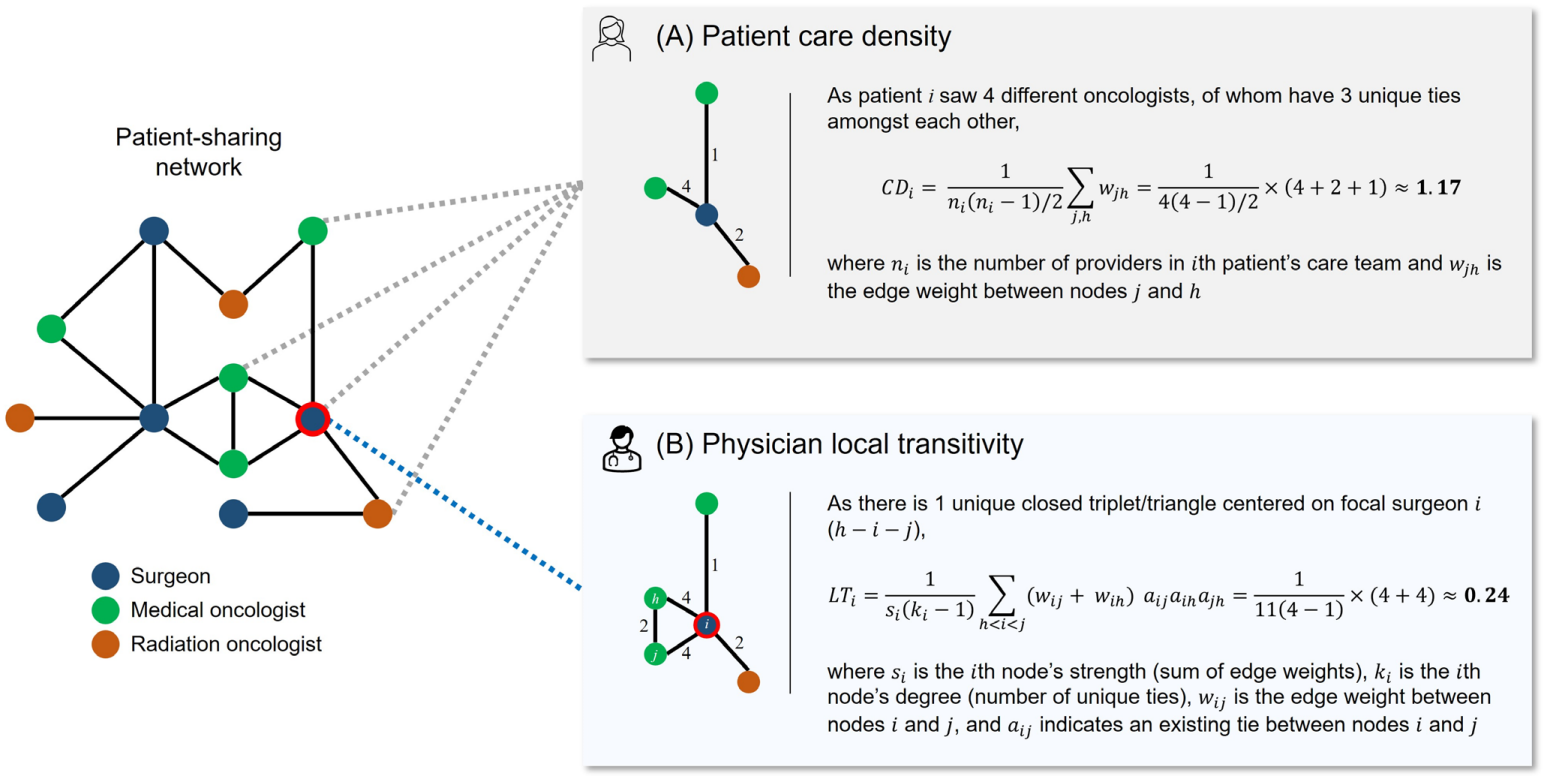
Illustration of the network measures of interest. (A) Over the course of their treatment, patient i received care from 4 physicians, of whom have 6 possible ties ($4\left[4-1\right]/2=6$). The existing ties between this set of physicians have edge weights that sum to 7 (4+2+1=7). Therefore, care density for patient i is calculated as 7/6≈1.17. (B) Surgeon i is directly connected to four other physicians in their egocentric network. There is one unique closed triplet centered on surgeon i (h−i−j). For this triplet, the strength (sum of edge weights) of nodes connected to surgeon i is 8 (4+4=8). The strength and degree (number of unique ties) of surgeon i are used to calculate the denominator, which is 33 ($11\left[4-1\right]=33$). Therefore, local transitivity for surgeon i is calculated as 8/33≈0.24.

The conceptual outcome of interest was care coordination, which was operationalized by the following measures computed on the nationwide patient‐sharing network (Table 1):

1. *Care density*: A patient‐level measure defined as the ratio of the sum of edge weights to the maximum number of possible ties among a patient's care team, with values ranging from [0, ∞) (undefined when there is one provider due to zero division) [19]. Care density reflects the extent of patient‐sharing among a patient's team of treating physicians. Patients with high care density are treated by physicians who share many patients among each other, indicating a high level of team familiarity, and are thought to be better positioned to receive coordinated care (Figure 1A).
2. *Local transitivity*: A physician‐level measure defined as the ratio of the strength (i.e., sum of edge weights) of closed triplets/triangles to the maximum strength of possible triplets/triangles centered on the physician, with values ranging from [0, 1] [28]. Local transitivity represents a physician's propensity to share patients with physicians who are also connected to each other (i.e., a friend of a friend is a friend of mine). Physicians with high local transitivity often form cohesive clusters/cliques and are thought to be better positioned to provide coordinated care (Figure 1B).

**TABLE 1:** Network measures of interest.

Measure	Assignment	Definition	Derivation	Interpretation	Range
Care density	Patient	The ratio of the sum of edge weights to the maximum number of possible ties among a patient's care team	$CD_{i}=\frac{1}{n_{i}\left(n_{i}-1\right)/2}\sum_{j,h}w_{\mathrm{jh}}$ where $n_{i}$ is the number of providers in the i th patient's care team and $w_{\mathrm{jh}}$ is the edge weight between nodes j and h	The level of familiarity among a patient's team of treating physicians	[0, ∞)
Local transitivity^a^	Physician	The ratio of the strength of closed triplets/triangles to the maximum strength of possible triplets centered on a physician	$LT_{i}=\frac{1}{s_{i}\left(k_{i}-1\right)}\sum_{h<i<j}\left(w_{\mathrm{ij}}+w_{\mathrm{ih}}\right)a_{\mathrm{ij}}a_{\mathrm{ih}}a_{\mathrm{jh}}$ where $s_{i}$ is the i th node's strength (sum of edge weights), $k_{i}$ is the i th node's degree (number of unique ties), $w_{\mathrm{ij}}$ is the edge weight between nodes i and j, and $a_{\mathrm{ij}}$ indicates the existence of a tie between nodes i and j	The physician's propensity to form cohesive clusters/cliques with their peers	[0, 1]

^a^ Calculated by the methodology proposed in Barrat et al. [28], which considers weighted edges in its calculation.

Because care density and local transitivity are patient‐ and physician‐level measures, respectively, we aggregated these measures to HRRs by attributing patients and physicians to one HRR (based on the plurality of their claims) and computing the medians of the resulting distributions. We multiplied local transitivity by 100 to scale it to a similar range as care density prior to analysis.

### 2.8 Statistical Analysis

We used one‐way analysis of variance to assess the independent associations between HRR attributes and oncology outreach tertiles by oncology specialty. In primary analysis, we used generalized linear models to examine the retrospective associations between HRR oncology outreach tertiles computed in 2016–2017 and HRR care coordination measures computed in 2018–2019 for each oncology specialty to preclude mechanical correlations among the exposure and outcome variables. Based on the distributions of the outcome variables (Figure S1B), we modeled care density with a gamma family and local transitivity with a gaussian family, using a log‐link in both models to enable a multiplicative interpretation of the estimates. We adjusted for all HRR demographic and oncologist per capita variables to account for potential confounding related to population characteristics and supply/demand. We also included an additional covariate for the number of encounters during the exposure period to account for cancer care utilization rates within each HRR. In sensitivity analysis, we explored how the estimates changed when modeling 1‐year lags (instead of 2‐year lags) between the exposure and outcome variables using hierarchical models with a random intercept for HRR. As a post hoc analysis, we investigated if HRR rurality (approximated using the proportion of rural patients) modified the primary associations, accounting for multiple testing via a Bonferroni correction (9 tests per outcome: *p* = 0.05/9 ≈ 0.006). In each statistical model, we reported exponentiated point estimates (multiplicative effects) and 95% confidence intervals, along with corresponding *p* values. All statistical tests were two sided and assumed a Type I error of 0.05.

## 3 Results

We identified 344,825 incident cancer patients who met our study inclusion criteria. The study cohort comprised 198,952 (57.7%) patients with breast cancer, 78,404 (22.7%) patients with colorectal cancer, and 67,469 (19.6%) patients with lung cancer. Of these patients, a large majority were White (≈90%) and lived in urban areas (≈80%) (Table S3). Over the study period, 33,686 oncologists provided care to patients in the study cohort, of whom 16,672 (49.5%) were surgeons, 12,309 (36.5%) were medical oncologists, and 4705 (14.0%) were radiation oncologists.

### 3.1 Distributions of HRR Attributes Across Oncology Outreach Tertiles

**FIGURE 2:**
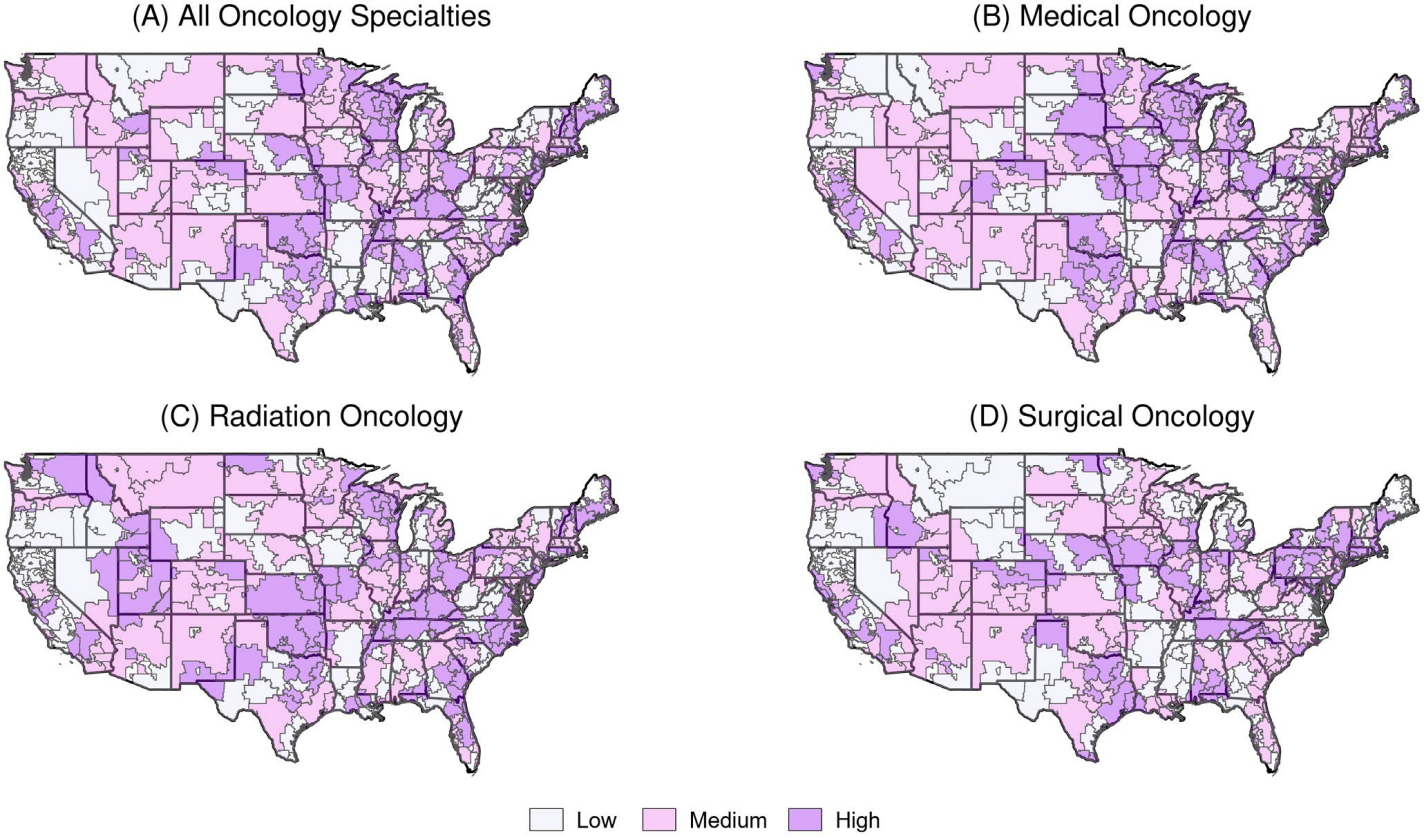
Distribution of oncology outreach tertiles across non‐continental US HRRs, faceted by oncology specialty.

Using encounter data from 2016–2017, we calculated the proportion of outreach visits by oncology specialty for each HRR and binned them into tertiles. The distributions of select HRR attributes varied significantly across medical oncology outreach tertiles. Specifically, the proportion of low‐income beneficiaries was greater in HRRs with lower medical oncology outreach (*p* < 0.01); the number of medical oncologists per capita was greatest in HRRs with medium medical oncology outreach, followed by HRRs with high and low medical oncology outreach (*p* < 0.01); and the number of radiation oncologists per capita was greater in HRRs with higher medical oncology outreach (*p* < 0.01). The same HRR attributes also varied significantly across radiation oncology outreach tertiles. Specifically, the proportion of low‐income beneficiaries was greatest in HRRs with low radiation oncology outreach, followed by HRRs with high and medium radiation oncology outreach (*p* = 0.03); the number of medical oncologists per capita was greatest in HRRs with medium radiation oncology outreach, followed by HRRs with high and low radiation oncology outreach (*p* = 0.01); and the number of radiation oncologists per capita was greater in HRRs with higher radiation oncology outreach (*p* < 0.01). Lastly, the distributions of two HRR attributes varied across surgical oncology outreach tertiles. These included the proportion of male beneficiaries and the proportion of rural beneficiaries, which were greater in HRRs with lower surgical oncology outreach (*p* = 0.01 and *p* < 0.01, respectively) (Table 2). Finally, we observed that the amount and type of oncology outreach appeared to vary substantially by HRR when visualized (Figure 2).

**TABLE 2:** Distribution of HRR attributes across oncology outreach tertiles by oncology specialty.

	Medical oncology outreach				Radiation oncology outreach				Surgical oncology outreach			
	Low	Med	High	*p*	Low	Med	High	*p*	Low	Med	High	*p*
Demographics, % (SD)												
Black beneficiaries	7.17 (8.06)	7.37 (9.08)	6.91 (8.05)	0.93	7.46 (9.38)	6.58 (7.69)	7.41 (8.03)	0.70	7.31 (9.56)	7.64 (7.96)	6.50 (7.51)	0.61
Hispanic beneficiaries	6.19 (12.9)	5.77 (9.69)	3.46 (4.84)	0.10	6.51 (12.5)	4.56 (7.90)	4.34 (8.13)	0.22	4.69 (9.74)	4.77 (8.30)	5.98 (11.1)	0.57
Low‐income beneficiaries	9.67 (3.07)	9.11 (2.70)	8.52 (2.00)	0.01	9.67 (3.09)	8.72 (2.27)	8.92 (2.50)	0.03	9.58 (2.82)	8.88 (2.24)	8.85 (2.85)	0.09
Male beneficiaries	45.0 (1.34)	44.9 (1.48)	44.9 (1.34)	0.78	45.0 (1.36)	45.0 (1.46)	44.8 (1.33)	0.48	45.2 (1.59)	45.0 (1.32)	44.6 (1.17)	0.01
Medicaid‐eligible beneficiaries	9.22 (4.88)	9.72 (4.98)	8.77 (3.13)	0.31	9.69 (4.63)	8.96 (4.22)	9.06 (4.40)	0.44	9.29 (4.27)	8.71 (3.52)	9.71 (5.28)	0.27
Rural beneficiaries	24.6 (21.3)	25.5 (24.9)	29.6 (27.0)	0.31	27.5 (24.2)	24.8 (23.8)	27.4 (25.7)	0.67	35.5 (27.4)	24.3 (22.1)	19.8 (21.1)	< 0.01
Oncologists per capita, *N* (SD)^a^												
Medical oncology	2.62 (1.28)	3.73 (1.46)	3.52 (1.54)	< 0.01	2.93 (1.42)	3.55 (1.51)	3.38 (1.53)	0.01	3.09 (1.64)	3.36 (1.56)	3.41 (1.28)	0.26
Radiation oncology	1.18 (0.88)	1.49 (0.70)	1.59 (1.08)	< 0.01	0.96 (0.62)	1.62 (0.68)	1.69 (1.15)	< 0.01	1.41 (1.15)	1.40 (0.75)	1.45 (0.79)	0.94
Surgical oncology	5.10 (2.00)	4.98 (1.51)	4.87 (1.84)	0.65	5.18 (1.84)	4.73 (1.69)	5.04 (1.83)	0.18	5.03 (1.87)	4.93 (1.79)	4.99 (1.73)	0.93

^a^ Calculated as the ratio of oncologists per every 100 study cohort patients.

### 3.2 Associations Between Oncology Outreach Tertiles and Care Coordination Measures

Using the nationwide network constructed from 2018–2019 claims, we calculated the network measures of interest and aggregated them to the HRR level. Among HRRs, median care density was 9.3 (IQR: 6.9–13.0) and median local transitivity was 50.1 (IQR: 45.3–54.5). The distribution of care density appeared to be right skewed, whereas the distribution of local transitivity was approximately normal (Figure S1B).

**FIGURE 3:**
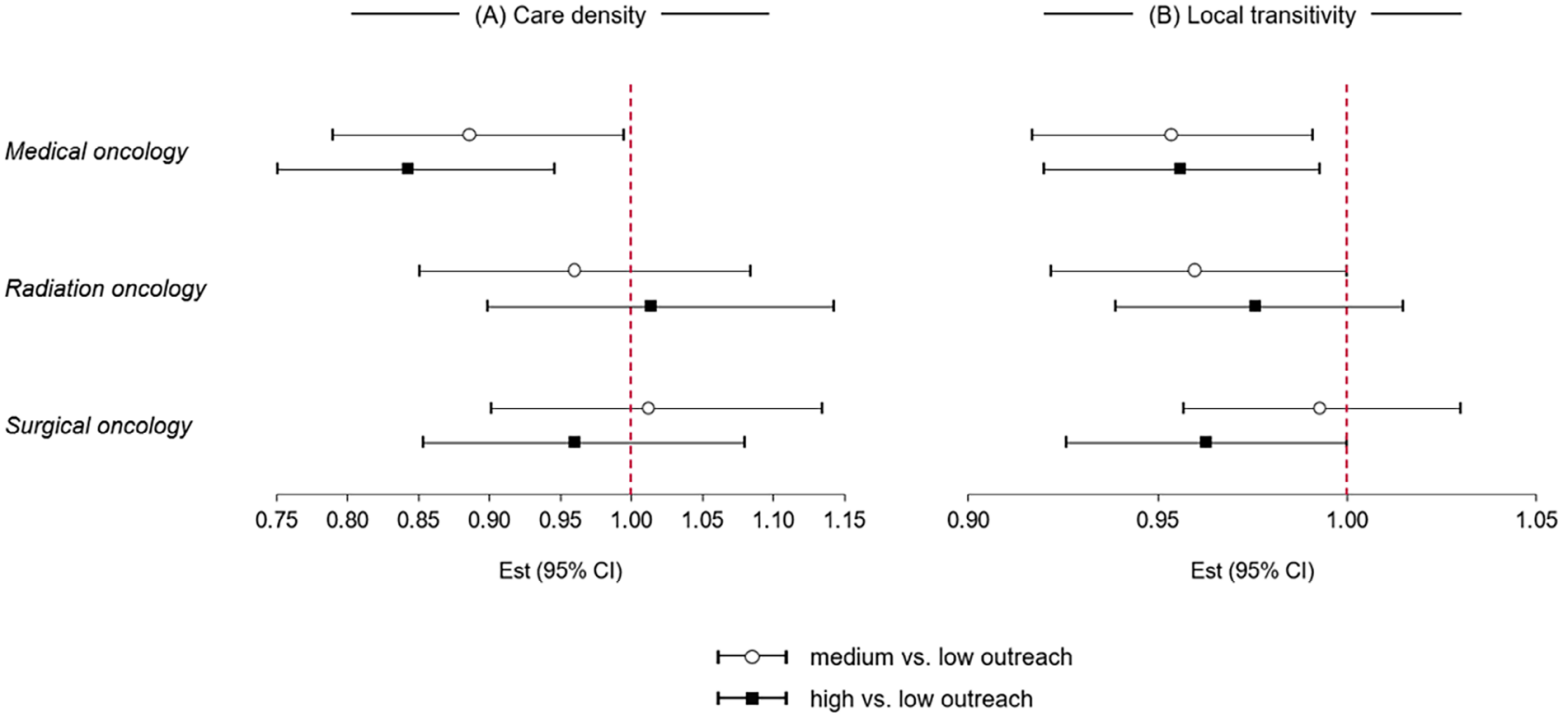
Adjusted associations between oncology outreach and care coordination measures by oncology specialty. (A) HRRs with high and medium medical oncology outreach were associated with 16% (95% CI: 5–25) and 11% (95% CI: 0–21) decreases in care density, respectively, compared to HRRs with low medical oncology outreach. (B) HRRs with high and medium medical oncology outreach were associated with 4% (95% CI: 1–8) and 5% (95% CI: 1–8) decreases in local transitivity, respectively, compared to HRRs with low medical oncology outreach.

In adjusted analysis, we found that HRRs with high medical oncology outreach were associated with 16% decrease in care density (95% CI: 5–25) and 4% decrease in local transitivity (95% CI: 1–8) compared to HRRs with low medical oncology outreach. We also found that HRRs with medium medical oncology outreach were associated with 11% decrease in care density (95% CI: 0–21) and 5% decrease in local transitivity (95% CI: 1–8) compared to HRRs with low medical oncology outreach. We did not observe any statistically significant results when considering the associations between care coordination measures with radiation and surgical oncology outreach (Figure 3, Table S4).

Our results were largely consistent when exploring 1‐year lags between exposure and outcome variables during sensitivity analysis. Although the medical oncology outreach point estimates remained statistically significant, they attenuated under this approach. We also observed a new statistically significant effect between high surgical oncology outreach and local transitivity (Table S5). Given the reduced stability of network measures calculated in annual versus biannual periods, these results are thought to be less reliable and instead serve as confirmatory estimates of the detected effects.

In post hoc analysis, we found no evidence that HRR rurality modified the associations between oncology outreach and care coordination measures (Table 3).

**TABLE 3:** Post hoc tests of HRR rurality as an effect modifier of the primary associations^a^ ^,^ ^b^ ^,^ ^c^.

	Care density		Local transitivity	
	Est (95% CI)	*p*	Est (95% CI)	*p*
Medical oncology outreach × HRR rurality				
Low	1.01 (0.97, 1.05)	0.78	1.01 (1.00, 1.02)	0.24
Medium	1.03 (0.98, 1.08)	0.26	1.00 (0.99, 1.02)	0.89
High	1.03 (0.99, 1.08)	0.19	1.00 (0.98, 1.01)	0.60
Radiation oncology outreach × HRR rurality				
Low	1.04 (1.00, 1.07)	0.04	1.01 (1.00, 1.02)	0.18
Medium	0.99 (0.94, 1.03)	0.52	1.00 (0.99, 1.02)	0.58
High	0.96 (0.92, 1.01)	0.09	0.99 (0.98, 1.00)	0.12
Surgical oncology outreach × HRR rurality				
Low	1.00 (0.97, 1.03)	0.75	1.00 (0.99, 1.01)	0.47
Medium	1.02 (0.98, 1.07)	0.36	1.01 (1.00, 1.03)	0.06
High	1.06 (1.01, 1.11)	0.02	1.01 (1.00, 1.03)	0.08

^a^ Separate models were fit for each set of interactions (by oncology specialty), adjusting for all HRR demographic and oncologist per capita variables, as well as the number of encounters in the exposure period.

^b^ Exponentiated point estimates and 95% confidence intervals are presented.

^c^ HRR rurality was approximated by the proportion of rural patients, with units operationalized as 10% increments.

## 4 Discussion

In this study, we analyzed associations between HRR oncology outreach tertiles and care coordination outcomes. We found that HRRs with high and medium medical oncology outreach were associated with reduced care density and local transitivity compared to HRRs with low oncology outreach. To our knowledge, this is the first study to explore associations between oncology outreach and network‐based measures of care coordination.

The inverse associations between higher medical oncology outreach and lower care coordination measures can be largely explained by the increased rates of medical oncologists commuting to satellite locations. As approximately 97% of oncologists maintain primary practice locations in metropolitan areas [1, 29, 30], which often cluster toward the middle of centralized/dense health systems [31], we expected the general reallocation of oncology resources to disrupt patient‐sharing relationships maintained in these regionalized settings. This was particularly hypothesized to be true for medical oncologists, as they often serve as the primary point of contact for initiating, referring, and overseeing cancer care [32, 33, 34]; therefore, their travel from high‐volume regionalized clinics to satellite clinics could create challenges in cancer care teaming and coordination. We also postulate that physician relationships in high medical oncology outreach markets may be somewhat fragmented at satellite locations due to a lack of co‐located radiation oncologists and surgeons. This is reasonable as oncology outreach policies have historically focused on extending medical oncology services specifically [5, 8, 35, 36, 37], so we do not necessarily expect medical oncology outreach arrangements to always be deployed in conjunction with radiation and surgical oncology outreach arrangements. Together, both factors may explain why we observed lower care coordination measures in markets with higher levels of medical oncology outreach.

In contrast, we did not observe reductions in care coordination measures in markets with high radiation or surgical oncology outreach. It is possible that many radiation oncologists and surgeons conduct outreach with the goal of establishing point‐of‐service before referring patients back to their primary locations for radiotherapy and surgery, respectively. Prior research showed that much of the oncology outreach provided by radiation oncologists and surgeons to breast cancer patients—the majority of the current sample—was composed of diagnostic or consultative services during the preoperative or postoperative phases of care [7]. After these appointments, patients may be funneled back to the attending's primary clinic for treatment, as the delivery of radiotherapy and surgery requires medical equipment that is often difficult to move along with additional staff who are typically concentrated at regionalized hospitals [5]. Under this model of care, we would not expect patient‐sharing patterns at their primary locations to observe significant fragmentation, as cancer treatment is still physically coordinated and provided there.

Despite linking medical oncology outreach with decreased care coordination, we believe the benefits of the outreach model outweigh this drawback. Such benefits include increased accessibility, timeliness, and utilization of cancer care for underserved, and typically rural patient populations [4, 5, 6, 7, 8]. Nonetheless, it is important to recognize the potential trade‐off between outreach and reduced care coordination, as lower coordination can impact patient quality measures, costs, and overall outcomes [16, 17, 18, 19, 20]. Health systems that elect to implement outreach models or those with active oncology outreach arrangements may consider coupling these initiatives with other policies to hedge against care fragmentation, specifically at regionalized centers where oncologist efforts are typically reallocated from. For example, patient navigation programs could be deployed in health systems with high medical oncology outreach to support the patients and teams of medical oncologists who regularly commute to underserved areas [12, 38, 39]. Teleoncology services and/or virtual tumor boards could also be leveraged to promote care continuity while oncologists are remote/traveling to satellite locations [40, 41]. Furthermore, innovative payment and delivery models, such as the Oncology Care Model, could be implemented in conjunction with outreach policies, which would incentivize health systems to uphold high standards of care coordination at both regionalized and satellite locations [42, 43].

Finally, in our post hoc analysis, we explored HRR rurality as an effect modifier of the inverse associations between medical oncology outreach and care coordination, finding no statistical evidence of any effect modification. Even though these results represent “null” findings, they show that the negative effects of medical oncology outreach on care coordination do not differ across markets with varying densities of rural patients; in other words, we expect these effects to be constant or homogeneous across all rural and urban areas. This information is useful for policymakers, as it implies that outreach arrangements can be implemented in any market without heightened or additional negative effects. While this analysis does not support rurality as an effect modifier, it does suggest that outreach arrangements are well suited in all markets. Extending this research to simulate the market‐specific or within‐system effects of outreach policies on care coordination and other outcomes (e.g., patient travel burden and physician utility) would be greatly beneficial for understanding where these policies may be most effective [44].

Our study has several limitations. First, our analyses are based on a sample of incident breast, colorectal, and lung cancer fee‐for‐service Medicare patients and their associated Part B claims; therefore, these findings may not generalize to less common cancer types or non‐Medicare populations. Second, we did not consider other physicians outside of oncology specialists in our patient‐sharing network (e.g., primary care physicians). As a result, these findings only provide insights into the coordination of cancer care once initiated, rather than the coordination of care along the entire patient journey. Third, our use of HRRs as proxies for large health systems is somewhat subjective and does not necessarily capture the nuances of vertically or horizontally integrated health systems within HRRs. This choice also exposes our analyses to potential ecological fallacies, so future studies may consider using alternative geographic units or analyzing patient‐ or physician‐level observations. Fourth, as we defined the exposures and outcomes across different time periods, we cannot rule out the possibility of misclassification bias. However, we postulate that any incurred misclassification bias is non‐differential in nature, resulting in more conservative results. Fifth, the reported associations are prone to unmeasured confounding, as we were unable to quantify certain HRR attributes that may distort the exposure–outcome relationships (e.g., competition and financial resources). Sixth, we did not explore the effects of oncology outreach on more tangible measures of care coordination (e.g., patient‐reported measures) or costs, as these outcomes were unavailable or beyond the scope of the current study, respectively. Lastly, we cannot derive any causal inferences due to our retrospective study design. A more intensive longitudinal or prospective study would be better suited for interrogating causality.

## 5 Conclusions

In this retrospective study of Medicare claims, we found that high medical oncology outreach markets were associated with reduced care density and local transitivity measures compared to low medical oncology outreach markets. These findings demonstrate that while medical oncology outreach is beneficial for increasing access to care for underserved patient populations, it may fragment care coordination across clinical settings. This information is critical for markets with active outreach arrangements or those considering outreach models, as these initiatives may need to be coupled with other policies or interventions that promote care coordination to offset their negative effects. Future simulation studies that explore the market‐specific or within‐system effects of oncology outreach would be greatly beneficial for understanding where these policies may be most effective. Network measures provide an emerging means to monitor the effects of healthcare policies, such as oncology outreach, and can be leveraged to evaluate the efficacy of such strategies.

## Author Contributions

**Bruno T. Scodari:** conceptualization (lead), formal analysis (lead), funding acquisition (supporting), investigation (lead), methodology (lead), project administration (lead), supervision (lead), validation (lead), visualization (lead), writing – original draft (lead), writing – review and editing (lead). **Andrew P. Schaefer:** data curation (supporting), methodology (supporting), project administration (supporting), resources (supporting), validation (supporting), writing – original draft (supporting). **Nirav S. Kapadia:** conceptualization (supporting), investigation (supporting), methodology (supporting), project administration (supporting), supervision (supporting), validation (supporting), visualization (supporting), writing – original draft (supporting), writing – review and editing (supporting). **A. James O'Malley:** conceptualization (supporting), funding acquisition (supporting), investigation (supporting), methodology (supporting), project administration (supporting), supervision (supporting), validation (supporting), visualization (supporting), writing – original draft (supporting), writing – review and editing (supporting). **Erika L. Moen:** conceptualization (supporting), data curation (lead), formal analysis (supporting), funding acquisition (lead), investigation (supporting), methodology (supporting), project administration (supporting), resources (lead), supervision (equal), validation (supporting), visualization (supporting), writing – original draft (supporting), writing – review and editing (supporting).

## Ethics Statement

Dartmouth College's institutional review board approved all study protocols and issued a waiver for obtaining informed consent from human subjects prior to commencement of the study.

## Conflicts of Interest

The authors declare no conflicts of interest.

## Supporting information


Appendix S1.

## Data Availability

The data used in this retrospective study are not publicly available due to a data use agreement with CMS.
