# Supplementary material for: Associations Between Oncology Outreach and Patient‐Sharing Measures of Care Coordination
Source: Cancer Med. 2024 Dec 10;13(23):e70489. doi: 10.1002/cam4.70489 (PMC11632124; doi:10.1002/cam4.70489)

| **Supplemental Table 1. CPT and ICD-10 codes used to identify study cohort** | | |
| --- | --- | --- |
| **Cohort** | **Procedure** | **Codes** |
| Breast | Diagnosis | ICD-10: C50.x  ICD-9: 174.xx, 233.0 |
|  | Biopsy | CPT: 10021, 10022, 19000, 19001, 19081, 19082, 19083, 19084, 19085, 19086, 19100, 19101, 19120, 19125, 19126, 19281, 19282, 19283, 19284, 19285, 19286, 19287, 19288  ICD-10: 0HBT0ZX, 0HBT3ZX, 0HBT4ZX, 0HBT7ZX, 0HBT8ZX, 0HBU0ZX, 0HBU3ZX, 0HBU4ZX, 0HBU7ZX, 0HBU8ZX, 0HBV0ZX, 0HBV3ZX, 0HBV4ZX, 0HBV7ZX, 0HBV8ZX |
| Colorectal | Diagnosis | ICD-10: C18.x, C19, C20  ICD-9: 153.0, 153.1, 153.2, 153.3, 153.4, 153.5, 153.6, 153.7, 153.8, 153.9, 154.0, 154.1, 159.0 |
|  | Biopsy | CPT: 45305, 45308, 45309, 45315, 45320, 45331, 45333, 45338, 45342, 45380,  45383, 45384, 45385, 44100  The following require same day CPT code 88305: G0121, G0105, 45378, 45379, 45386, 45387, 45391, 45392  ICD-10: 0DBE0ZX, 0DBE3ZX, 0DBE4ZX, 0DBE7ZX, 0DBE8ZX, 0DBF0ZX,  0DBF3ZX, 0DBF4ZX, 0DBF7ZX, 0DBF8ZX, 0DBG0ZX, 0DBG3ZX, 0DBG4ZX, 0DBG7ZX, 0DBG8ZX, 0DBK0ZX, 0DBK3ZX, 0DBK4ZX, 0DBK7ZX, 0DBK8ZX, 0DBL0ZX, 0DBL3ZX, 0DBL4ZX, 0DBL7ZX, 0DBL8ZX, 0DBM0ZX, 0DBM3ZX, 0DBM4ZX, 0DBM7ZX, 0DBM8ZX, 0DBN0ZX, 0DBN3ZX, 0DBN4ZX, 0DBN7ZX, 0DBN8ZX, 0DBP0ZX, 0DBP3ZX, 0DBP4ZX, 0DBP7ZX, 0DBP8ZX |
| Lung | Diagnosis | ICD-10: C34.x  ICD-9: 162.xx |
|  | Biopsy | CPT: 32400, 32402, 32405, 32604, 31623, 31624, 31625, 31628, 31629, 31640, 31632, 31633, 32096, 32097, 32098  ICD-10: 0BBC0ZX, 0BBC3ZX, 0BBC4ZX, 0BBC7ZX, 0BBC8ZX, 0BBD0ZX, 0BBD3ZX, 0BBD4ZX, 0BBD7ZX, 0BBD8ZX, 0BBF0ZX, 0BBF3ZX, 0BBF4ZX, 0BBF7ZX, 0BBF8ZX, 0BBG0ZX, 0BBG3ZX, 0BBG4ZX, 0BBG7ZX, 0BBG8ZX, 0BBH0ZX, 0BBH3ZX, 0BBH4ZX, 0BBH7ZX, 0BBH8ZX, 0BBJ0ZX, 0BBJ3ZX, 0BBJ4ZX, 0BBJ7ZX, 0BBJ8ZX, 0BBK0ZX, 0BBK3ZX, 0BBK4ZX, 0BBK7ZX, 0BBK8ZX, 0BBL0ZX, 0BBL3ZX, 0BBL4ZX, 0BBL7ZX, 0BBL8ZX, 0BBM0ZX, 0BBM3ZX, 0BBM4ZX, 0BBM7ZX, 0BBM8ZX |

| **Supplemental Table 2. Taxonomy codes used to identify physician specialty** | |
| --- | --- |
| Specialty | Codes |
| Medical oncology | 207RH0003X, 207RX0202X, 207VX0201X |
| Radiation oncology | 2085R0001X |
| Surgery^a^ | 2086X0206X, 208600000X, 2086S0122X, 208200000X, 208G00000X, 208C00000X |
| ^a^ Surgeons were also required to have at least one cancer-directed surgery claim associated with a patient in the study cohort | |

| **Supplemental Table 3. Patient characteristics, stratified by cancer type** | | | | |
| --- | --- | --- | --- | --- |
|  | Cancer type | | | |
|  | Breast | Colorectal | Lung | Combined |
|  | N=198952 | N=78404 | N=67469 | N=344825 |
| Age at diagnosis^a^ |  |  |  |  |
| 66-70 | 65982 (33.2%) | 20974 (26.8%) | 18803 (27.9%) | 105759 (30.7%) |
| 71-75 | 58295 (29.3%) | 20544 (26.2%) | 20198 (29.9%) | 99037 (28.7%) |
| 76-80 | 38901 (19.6%) | 17181 (21.9%) | 15741 (23.3%) | 71823 (20.8%) |
| >80 | 35774 (18.0%) | 19705 (25.1%) | 12727 (18.9%) | 68206 (19.8%) |
| Charlson comorbidities^b^ |  |  |  |  |
| 0 | 112458 (56.5%) | 30739 (39.2%) | 15290 (22.7%) | 158487 (46.0%) |
| 1 | 44133 (22.2%) | 18345 (23.4%) | 19884 (29.5%) | 82362 (23.9%) |
| 2+ | 42361 (21.3%) | 29320 (37.4%) | 32295 (47.9%) | 103976 (30.2%) |
| Low-income^c^ |  |  |  |  |
| No | 173768 (87.3%) | 66908 (85.3%) | 58135 (86.2%) | 298811 (86.7%) |
| Yes | 25184 (12.7%) | 11496 (14.7%) | 9334 (13.8%) | 46014 (13.3%) |
| Metastatic disease^d^ |  |  |  |  |
| No | 190603 (95.8%) | 67767 (86.4%) | 53573 (79.4%) | 311943 (90.5%) |
| Yes | 8349 (4.2%) | 10637 (13.6%) | 13896 (20.6%) | 32882 (9.5%) |
| Race^e^ |  |  |  |  |
| Minority | 20557 (10.3%) | 8679 (11.1%) | 6146 (9.1%) | 35382 (10.3%) |
| White | 178395 (89.7%) | 69725 (88.9%) | 61323 (90.9%) | 309443 (89.7%) |
| Rurality^f^ |  |  |  |  |
| Isolated | 8162 (4.1%) | 3732 (4.8%) | 3092 (4.6%) | 14986 (4.3%) |
| Small Rural | 10805 (5.4%) | 5162 (6.6%) | 4278 (6.3%) | 20245 (5.9%) |
| Large Rural | 20612 (10.4%) | 9406 (12.0%) | 7722 (11.4%) | 37740 (10.9%) |
| Urban | 159373 (80.1%) | 60104 (76.7%) | 52377 (77.6%) | 271854 (78.8%) |
| Sex^e^ |  |  |  |  |
| Male | 0 (0%) | 38210 (48.7%) | 30395 (45.1%) | 68605 (19.9%) |
| Female | 198952 (100%) | 40194 (51.3%) | 37074 (54.9%) | 276220 (80.1%) |
| ^a^ Estimated by patient age at the time of biopsy  ^b^ Assigned using the method presented in Klabunde et al., 2018  ^c^ Classified as living in a ZIP code with >20% of the population below the poverty line per the 2015-19 American Community Survey 5-year Estimates file  ^d^ Defined as having a diagnosis code for secondary malignancy within 90 days of biopsy  ^e^ Obtained from the CMS Master Beneficiary Summary file  ^f^ Determined using the four-tiered classification of secondary Rural-Urban Commuting Area codes according to the US Department of Agriculture’s Economic Research Service | | | | |

| **Supplemental Table 4. Adjusted associations between oncology outreach and care coordination measures by oncology specialty^a^** | | | | | | | | | | | | | | | | | | | |
| --- | --- | --- | --- | --- | --- | --- | --- | --- | --- | --- | --- | --- | --- | --- | --- | --- | --- | --- | --- |
|  | | Medical oncology outreach | | | | | | | Radiation oncology outreach | | | | | | Surgical oncology outreach | | | | |
|  | | Care density | | | | Local transitivity | | | Care density | | | Local transitivity | | | Care density | | | Local transitivity | |
|  | | Est  (95% CI) | | *P* | | Est (95% CI) | *P* | | Est (95% CI) | *P* | | Est  (95% CI) | *P* | | Est  (95% CI) | *P* | | Est  (95% CI) | *P* |
| Oncology outreach level | |  | |  | |  |  | |  |  | |  |  | |  |  | |  |  |
| Medium vs. low | | 0.89 (0.79, 1.00) | | 0.04 | | 0.95 (0.92, 0.99) | 0.02 | | 0.96 (0.85, 1.08) | 0.51 | | 0.96 (0.92, 1.00) | 0.05 | | 1.01 (0.90, 1.14) | 0.84 | | 0.99 (0.96, 1.03) | 0.70 |
| High vs. low | | 0.84 (0.75, 0.95) | | 0.01 | | 0.96 (0.92, 0.99) | 0.02 | | 1.01 (0.90, 1.14) | 0.82 | | 0.98 (0.94, 1.02) | 0.23 | | 0.96 (0.85, 1.08) | 0.50 | | 0.96 (0.93, 1.00) | 0.05 |
| Demographics, in 10% units | |  | |  | |  |  | |  |  | |  |  | |  |  | |  |  |
| Black beneficiaries | | 1.00 (0.92, 1.09) | | 0.99 | | 1.00 (0.98, 1.03) | 0.83 | | 0.99 (0.91, 1.08) | 0.78 | | 1.00 (0.97, 1.03) | 0.98 | | 0.99 (0.91, 1.08) | 0.87 | | 1.00 (0.97, 1.03) | 0.86 |
| Hispanic beneficiaries | | 1.03 (0.96, 1.11) | | 0.41 | | 1.02 (1.00, 1.05) | 0.11 | | 1.03 (0.95, 1.11) | 0.51 | | 1.02 (0.99, 1.05) | 0.14 | | 1.03 (0.96, 1.11) | 0.45 | | 1.02 (0.99, 1.04) | 0.16 |
| Low-income beneficiaries | | 0.86 (0.62, 1.19) | | 0.36 | | 0.94 (0.84, 1.05) | 0.29 | | 0.96 (0.69, 1.33) | 0.81 | | 0.97 (0.87, 1.08) | 0.56 | | 0.91 (0.65, 1.27) | 0.58 | | 0.95 (0.86, 1.06) | 0.38 |
| Male beneficiaries | | 0.97 (0.61, 1.56) | | 0.91 | | 0.99 (0.84, 1.16) | 0.91 | | 1.05 (0.65, 1.70) | 0.84 | | 1.00 (0.85, 1.17) | 0.97 | | 0.98 (0.60, 1.59) | 0.93 | | 0.93 (0.79, 1.09) | 0.39 |
| Medicaid-eligible beneficiaries | | 0.78 (0.65, 0.93) | | 0.01 | | 0.92 (0.87, 0.98) | 0.01 | | 0.76 (0.63, 0.91) | 0.01 | | 0.91 (0.86, 0.97) | 0.01 | | 0.77 (0.64, 0.92) | 0.01 | | 0.92 (0.87, 0.98) | 0.01 |
| Rural beneficiaries | | 1.03 (1.00, 1.05) | | 0.02 | | 1.01 (1.00, 1.01) | 0.12 | | 1.02 (1.00, 1.04) | 0.13 | | 1.00 (1.00, 1.01) | 0.24 | | 1.02 (0.99, 1.04) | 0.18 | | 1.00 (1.00, 1.01) | 0.36 |
| Oncologists per capita^b^ | |  | |  | |  |  | |  |  | |  |  | |  |  | |  |  |
| Medical oncology | | 0.38 (0.27, 0.55) | | 0.01 | | 0.89 (0.78, 1.01) | 0.07 | | 0.35 (0.25, 0.50) | 0.01 | | 0.85 (0.75, 0.96) | 0.01 | | 0.36 (0.26, 0.52) | 0.01 | | 0.86 (0.77, 0.97) | 0.02 |
| Radiation oncology | | 0.49 (0.28, 0.86) | | 0.01 | | 0.65 (0.53, 0.80) | 0.01 | | 0.45 (0.25, 0.84) | 0.01 | | 0.71 (0.58, 0.88) | 0.01 | | 0.45 (0.25, 0.80) | 0.01 | | 0.65 (0.53, 0.79) | 0.01 |
| Surgical oncology | | 0.41 (0.30, 0.54) | | 0.01 | | 1.18 (1.07, 1.30) | 0.01 | | 0.40 (0.30, 0.53) | 0.01 | | 1.17 (1.06, 1.28) | 0.01 | | 0.38 (0.29, 0.50) | 0.01 | | 1.19 (1.09, 1.30) | 0.01 |
| Other adjustments | |  | |  | |  |  | |  |  | |  |  | |  |  | |  |  |
| Number of encounters^c^ | | 1.01 (1.00, 1.02) | | 0.09 | | 1.00 (0.99, 1.00) | 0.04 | | 1.01 (0.99, 1.02) | 0.43 | | 0.99 (0.99, 1.00) | 0.01 | | 0.99 (0.97, 1.01) | 0.30 | | 0.99 (0.98, 0.99) | 0.01 |
| ^a^ Exponentiated point estimates and 95% confidence intervals are presented  ^b^ Calculated as the ratio of oncologists per every 100 study cohort patients  ^c^ Units in thousands | | | | | | | | | | | | | | | | | | | |
| **Supplemental Table 5. Sensitivity analysis of the associations between oncology outreach and care coordination measures by oncology specialty^a,b^** | | | | | | | | | | | | | | | | | | | |
|  | Medical oncology outreach | | | | | | | Radiation oncology outreach | | | | | | Surgical oncology outreach | | | | | |
|  | Care density | | | | Local transitivity | | | Care density | | | Local transitivity | | | Care density | | | Local transitivity | | |
|  | Est  (95% CI) | | *P* | | Est  (95% CI) | | *P* | Est  (95% CI) | | *P* | Est  (95% CI) | | *P* | Est  (95% CI) | | *P* | Est  (95% CI) | | *P* |
| Oncology outreach level |  | |  | |  | |  |  | |  |  | |  |  | |  |  | |  |
| Medium vs. low | 0.95 (0.91, 0.98) | | 0.01 | | 0.96 (0.93, 0.98) | | 0.01 | 1.00 (0.96, 1.04) | | 0.89 | 0.98 (0.96, 1.01) | | 0.14 | 1.01 (0.98, 1.05) | | 0.50 | 0.99 (0.97, 1.02) | | 0.47 |
| High vs. low | 0.95 (0.91, 0.99) | | 0.02 | | 0.96 (0.94, 0.99) | | 0.01 | 0.99 (0.95, 1.04) | | 0.74 | 0.98 (0.96, 1.01) | | 0.17 | 0.98 (0.94, 1.02) | | 0.23 | 0.97 (0.95, 1.00) | | 0.02 |
| Demographics, in 10% units |  | |  | |  | |  |  | |  |  | |  |  | |  |  | |  |
| Black beneficiaries | 0.99 (0.90, 1.10) | | 0.89 | | 0.99 (0.97, 1.00) | | 0.12 | 0.99 (0.90, 1.09) | | 0.86 | 0.99 (0.97, 1.00) | | 0.13 | 0.99 (0.90, 1.09) | | 0.85 | 0.98 (0.97, 1.00) | | 0.08 |
| Hispanic beneficiaries | 1.04 (0.95, 1.13) | | 0.43 | | 1.02 (1.01, 1.04) | | 0.01 | 1.03 (0.95, 1.13) | | 0.48 | 1.02 (1.01, 1.04) | | 0.01 | 1.04 (0.95, 1.13) | | 0.44 | 1.02 (1.01, 1.04) | | 0.01 |
| Low-income beneficiaries | 0.85 (0.58, 1.24) | | 0.39 | | 1.05 (0.98, 1.13) | | 0.16 | 0.87 (0.60, 1.28) | | 0.48 | 1.07 (1.00, 1.15) | | 0.07 | 0.86 (0.58, 1.26) | | 0.44 | 1.05 (0.98, 1.13) | | 0.14 |
| Male beneficiaries | 0.97 (0.56, 1.69) | | 0.92 | | 0.99 (0.89, 1.09) | | 0.77 | 0.98 (0.56, 1.70) | | 0.93 | 0.99 (0.89, 1.09) | | 0.79 | 0.96 (0.55, 1.68) | | 0.88 | 0.93 (0.84, 1.03) | | 0.15 |
| Medicaid-eligible beneficiaries | 0.84 (0.68, 1.03) | | 0.09 | | 0.88 (0.84, 0.91) | | 0.01 | 0.84 (0.68, 1.03) | | 0.09 | 0.87 (0.84, 0.91) | | 0.01 | 0.83 (0.68, 1.03) | | 0.09 | 0.88 (0.84, 0.91) | | 0.01 |
| Rural beneficiaries | 0.00 (0.00, 0.00) | | 0.01 | | 0.00 (0.00, 0.00) | | 0.01 | 0.00 (0.00, 0.00) | | 0.01 | 0.00 (0.00, 0.00) | | 0.01 | 0.00 (0.00, 0.00) | | 0.01 | 0.00 (0.00, 0.00) | | 0.01 |
| Oncologists per capita^c^ |  | |  | |  | |  |  | |  |  | |  |  | |  |  | |  |
| Medical oncology | 0.42 (0.28, 0.63) | | 0.01 | | 0.82 (0.75, 0.89) | | 0.01 | 0.41 (0.27, 0.62) | | 0.01 | 0.79 (0.73, 0.86) | | 0.01 | 0.41 (0.27, 0.62) | | 0.01 | 0.81 (0.75, 0.87) | | 0.01 |
| Radiation oncology | 0.52 (0.27, 1.02) | | 0.06 | | 0.76 (0.67, 0.86) | | 0.01 | 0.51 (0.26, 1.01) | | 0.05 | 0.80 (0.70, 0.91) | | 0.01 | 0.51 (0.26, 1.00) | | 0.05 | 0.75 (0.67, 0.85) | | 0.01 |
| Surgical oncology | 0.46 (0.33, 0.64) | | 0.01 | | 1.26 (1.18, 1.34) | | 0.01 | 0.46 (0.33, 0.63) | | 0.01 | 1.24 (1.17, 1.32) | | 0.01 | 0.45 (0.32, 0.63) | | 0.01 | 1.25 (1.18, 1.33) | | 0.01 |
| Other adjustments |  | |  | |  | |  |  | |  |  | |  |  | |  |  | |  |
| Number of encounters^d^ | 1.01 (1.00, 1.02) | | 0.01 | | 1.00 (0.99, 1.00) | | 0.01 | 1.01 (1.00, 1.02) | | 0.12 | 0.99 (0.99, 1.00) | | 0.01 | 1.01 (0.99, 1.03) | | 0.44 | 0.97 (0.96, 0.98) | | 0.01 |
| Year of exposure |  | |  | |  | |  |  | |  |  | |  |  | |  |  | |  |
| 2017 vs. 2016 | 1.21 (1.18, 1.24) | | 0.01 | | 0.99 (0.97, 1.02) | | 0.46 | 1.21 (1.18, 1.24) | | 0.01 | 1.00 (0.97, 1.02) | | 0.65 | 1.22 (1.18, 1.25) | | 0.01 | 1.00 (0.98, 1.02) | | 0.82 |
| 2018 vs. 2016 | 1.43 (1.39, 1.47) | | 0.01 | | 0.99 (0.97, 1.02) | | 0.62 | 1.44 (1.40, 1.48) | | 0.01 | 1.00 (0.97, 1.02) | | 0.81 | 1.44 (1.40, 1.49) | | 0.01 | 1.00 (0.98, 1.03) | | 0.84 |
| ^a^ Exponentiated point estimates and 95% confidence intervals are presented  ^b^ Care coordination measures computed in the following year were regressed onto oncology outreach levels computed in the previous year; hierarchical generalized linear models with random intercepts for HRR were used and year was controlled for as a fixed effect  ^c^ Calculated as the ratio of oncologists per every 100 study cohort patients  ^d^ Units in thousands | | | | | | | | | | | | | | | | | | | |

**Supplemental Figure 1. Distributions of pooled oncology outreach for all specialties and care coordination network outcomes**

Linchpin score (scaled)

Degree centr. (scaled)

Care density

Linchpin score (scaled)

Degree centr. (scaled)

Care density


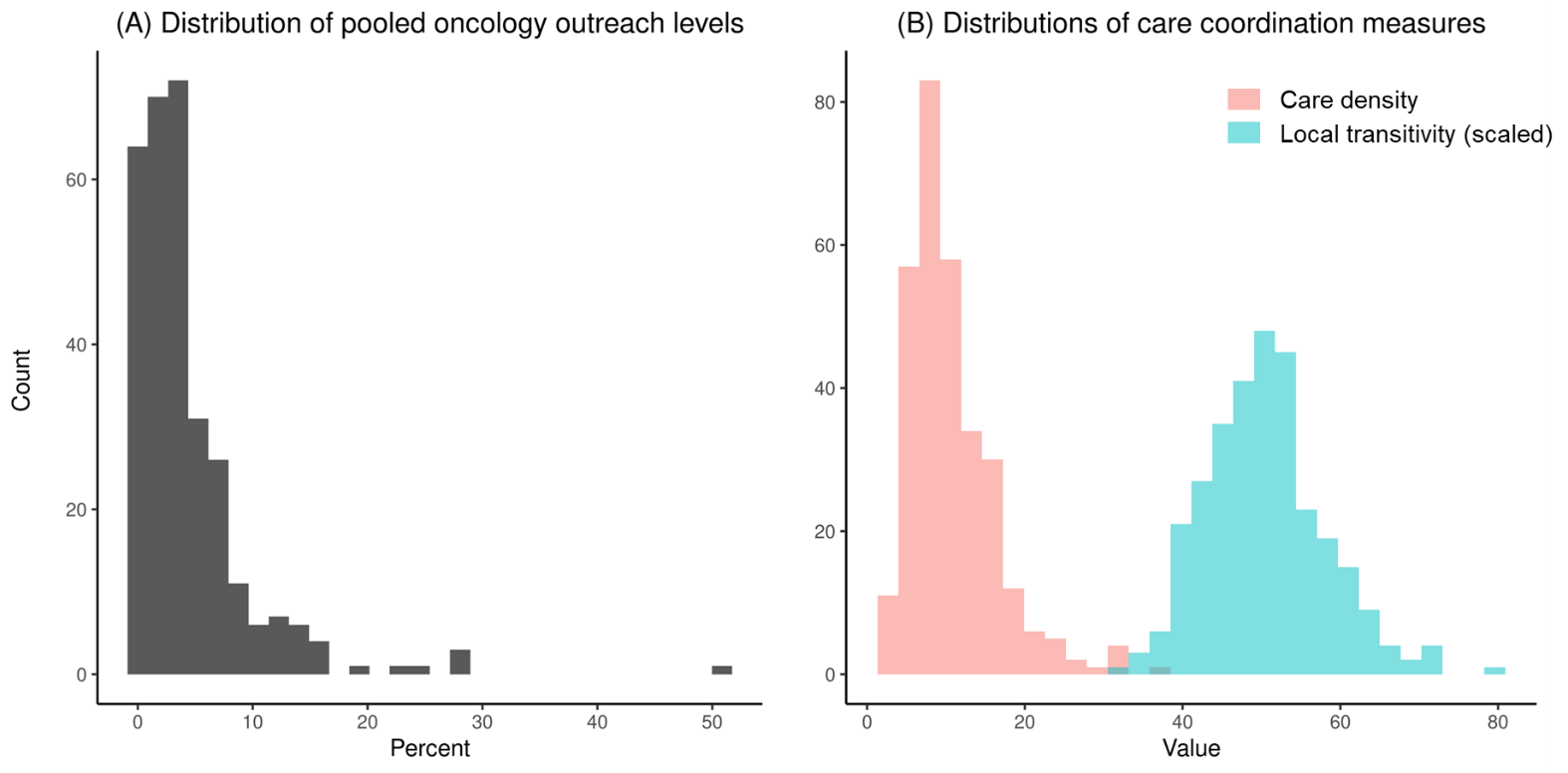

Supplement: Supplementary file 1 — Appendix S1. [file CAM4-13-e70489-s001.docx]
